# Supplementary material for: The Influence of Maternal Information Sources on Infant Oral Hygiene Practices for Six-Month-Olds in South Australia: A Cross-Sectional Study
Source: Int J Environ Res Public Health. 2025 May 23;22(6):826. doi: 10.3390/ijerph22060826 (PMC12192995; doi:10.3390/ijerph22060826)
Supplement: Supplementary file 1 [file ijerph-22-00826-s001.zip › ijerph-3580920-supplementary.pdf]

Supplementary material

SUPPLEMENTARY Table S1

Results from Multivariable Logistic Regression Model (Unadjusted and Adjusted) on Gum/Tooth Cleaning for 6-month-old Infants in the Past 3 Months

Variable	Infant gum/tooth cleaning in the past 3 months (%)	Unadjusted			Adjusted		
		Odds Ratio	95% CI	P-value	Odds Ratio	95% CI	P-value
All maternal characteristics							
IRSAD							
Deciles 1-2	141/311 (45.3%)	1 (reference)					
Deciles 3-5	179/475 (37.7%)	0.73	0.55, 0.97	**0.03**	0.73	0.53, 0.99	**0.047**
Deciles 6-8	135/371 (36.4%)	0.69	0.51, 0.94	**0.02**	0.73	0.52, 1.02	0.067
Deciles 9-10	87/266 (32.7%)	0.59	0.41, 0.82	**<0.01**	0.61	0.42, 0.90	**0.012**
Health care card							
Yes	156/366 (42.6%)	1 (reference)					
None	300/850 (35.3%)	0.73	0.57, 0.94	**0.02**	0.83	0.61, 1.11	0.212
Other	41/105 (39.0%)	0.86	0.55, 1.34	0.51	0.81	0.49, 1.32	0.387
FTB Part A Eligibility							
Yes	308/791 (38.9%)	1 (reference)					
No	84/220 (38.2%)	0.96	0.71, 1.32	0.84	1.09	0.76, 1.56	0.653
Unsure	150/418 (35.9%)	0.87	0.68, 1.12	0.29	1.03	0.77, 1.38	0.852
Private insurance							
Yes	266/729 (36.5%)	1 (reference)					
No	271/687 (39.4%)	1.13	0.91, 1.41	0.25	1.00	0.77, 1.29	0.996
Mother’s age at childbirth							
≤ 24 years	97/181 (53.6%)	1 (reference)					
25-34 years	353/952 (37.0%)	0.51	0.37, 0.70	**<0.01**	0.53	0.37, 0.77	**<0.01**
35+ years	95/300 (31.7%)	0.40	0.27, 0.59	**<0.01**	0.43	0.28, 0.67	**<0.01**
Mother’s birth country							
Australia, New Zealand, and UK	420/1,070 (39.3%)	1 (reference)					
Asia – other	51/160 (31.9%)	0.72	0.51, 1.03	0.07	0.32	0.15, 0.70	**<0.01**
Asia – India	38/107 (35.5%)	0.85	0.56, 1.29	0.45	0.47	0.20, 1.11	0.083
Other	33/89 (37.1%)	0.91	0.58, 1.43	0.69	0.56	0.28, 1.15	0.117
Mother’s primary language							
English	431/1,109 (38.9%)	1 (reference)					
Other	111/312 (35.6%)	0.86	0.66, 1.13	0.29	2.30	1.11, 4.77	**0.025**
Mother’s indigenous status							
No	536/1,409 (38.0%)	1 (reference)					
Yes	6/15 (40.0%)	1.08	0.38, 3.10	0.88	1.12	0.39, 3.22	0.838
Maternal education completed							
School/ vocational	290/676 (42.9%)	1 (reference)					

Variable	Infant gum/tooth cleaning in the past 3 months (%)	Unadjusted			Adjusted		
		Odds Ratio	95% CI	P-value	Odds Ratio	95% CI	P-value
Some university and above	255/754 (33.8%)	0.68	0.55, 0.84	**<0.01**	0.89	0.68, 1.17	0.400
Mother's work status							
Full-time	221/558 (39.6%)	1 (reference)					
Part-time	154/431 (35.7%)	0.85	0.65, 1.09	0.21	0.83	0.62, 1.10	0.199
Self-employed	16/57 (28.1%)	0.59	0.33, 1.08	0.09	0.65	0.34, 1.25	0.194
Unemployed/ home duties/ pensioner	150/380 (39.5%)	0.99	0.76, 1.29	0.96	0.77	0.57, 1.06	0.113
Source of information consulted							
No information consulted							
No	396/968 (40.1%)	1 (reference)					
Yes	152/471 (32.2%)	0.68	0.55, 0.87	**<0.01**	0.55	0.38, 0.80	**<0.01**
Child health nurse							
No	413/1,096 (37.6%)	1 (reference)					
Yes	135/343 (39.4%)	1.07	0.83, 1.38	0.58	0.95	0.69, 1.30	0.740
Doctor							
No	484/1,285 (37.6%)	1 (reference)					
Yes	64/154 (39.4%)	1.18	0.84, 1.65	0.35	1.19	0.81, 1.76	0.377
Dietician							
No	543/1,431 (37.9%)	1 (reference)					
Yes	5/8 (62.5%)	2.72	0.65, 11.45	0.17	1.27	0.30, 5.45	0.747
Dentist							
No	466/1,268 (36.7%)	1 (reference)					
Yes	82/171 (47.9%)	1.59	1.15, 2.19	**<0.01**	1.41	0.97, 2.05	0.069
Mother-in-law							
No	471/1,251 (37.6%)	1 (reference)					
Yes	77/188 (40.9%)	1.14	0.84, 1.57	0.38	0.77	0.51, 1.14	0.191
Other female personnel (e.g. play group mums)							
No	459/1,185 (38.7%)	1 (reference)					
Yes	89/254 (35.0%)	0.85	0.64, 1.13	0.27	0.73	0.52, 1.02	0.065
Information pamphlets/books							
No	422/1,086 (38.8%)	1 (reference)					
Yes	126/353 (35.7%)	0.87	0.68, 1.12	0.28	0.71	0.52, 0.97	**0.029**
Pharmacist†							
No	545/1,436 (37.9%)	1 (reference)					
Yes	3/3 (100%)	11.44	0.59, 221.90	0.11	8.93	0.45, 176.82	0.151
Other dental professional (e.g. dental hygienist)							
No	547/1,435 (38.1%)	1 (reference)					
Yes	1/4 (25.0%)	0.54	0.05, 5.22	0.59	0.66	0.10, 4.60	0.677
Internet							
No	521/1,373 (37.9%)	1 (reference)					
Yes	27/66 (40.1%)	1.13	0.68, 1.87	0.63	1.18	0.66, 2.11	0.583
Experience from previous child							

Variable	Infant gum/tooth cleaning in the past 3 months (%)	Unadjusted			Adjusted		
		Odds Ratio	95% CI	P-value	Odds Ratio	95% CI	P-value
No	534/1,415 (37.7%)	1 (reference)					
Yes	14/24 (58.3%)	2.31	1.01, 5.24	**0.04**	1.88	0.78, 4.50	0.159

Bold values represent statistical significance. CI, confidence interval.

† Firth's penalisation method applied.

SUPPLEMENTARY Table S2

Results from Multivariable Logistic Regression Analysis (Unadjusted and Adjusted) on the Frequency of Cleaning Primary Teeth in 6-Month-Old Infants

Variable	Infant tooth cleaning 2+ times per day (%)	Unadjusted			Adjusted		
		Odds Ratio	95% CI	P-value	Odds Ratio	95% CI	P-value
All maternal characteristics							
IRSAD							
Deciles 1-2	56/67 (83.6%)	1 (reference)					
Deciles 3-5	55/76 (72.4%)	0.51	0.23, 1.17	0.11	0.46	0.19, 1.14	0.094
Deciles 6-8	44/57 (77.2%)	0.56	0.27, 1.63	0.37	0.61	0.23, 1.60	0.317
Deciles 9-10	30/44 (68.2%)	0.42	0.17, 1.04	0.06	0.47	0.17, 1.27	0.135
Health care card							
Yes	68/82 (82.9%)	1 (reference)					
None	97/133 (72.9%)	0.55	0.28, 1.11	0.09	0.81	0.35, 1.89	0.629
Other	12/17 (70.6%)	0.49	0.15, 1.63	0.25	0.65	0.18, 2.42	0.525
FTB Part A Eligibility							
Yes	117/147 (79.6%)	1 (reference)					
No	25/34 (73.5%)	0.71	0.30, 1.68	0.44	1.07	0.40, 2.85	0.896
Unsure	44/63 (69.8%)	0.59	0.30, 1.16	0.13	0.76	0.35, 1.65	0.484
Private insurance							
Yes	82/114 (71.9%)	1 (reference)					
No	102/128 (79.7%)	1.53	0.85, 2.77	0.16	1.41	0.69, 2.87	0.347
Mother's age at childbirth							
≤ 24 years	29/39 (74.4%)	1 (reference)					
25-34 years	125/168 (74.4%)	1.00	0.45, 2.23	0.96	1.23	0.47, 3.22	0.666
35+ years	32/38 (84.2%)	1.84	0.59, 5.69	0.29	1.87	0.54, 6.41	0.320
Mother's birth country†							
Australia, New Zealand, and UK	155/207 (74.9%)	1 (reference)					
Asia – other	13/18 (72.2%)	0.83	0.29, 2.34	0.72	0.58	0.04, 8.10	0.684
Asia – India	7/9 (77.8%)	1.01	0.23, 4.38	0.99	0.61	0.02, 14.82	0.759
Other	11/11 (100%)	7.76	0.44, 134.10	0.16	4.61	0.09, 228.49	0.443
Mother's primary language							
English	156/208 (75.0%)	1 (reference)					
Other	30/37 (81.1%)	1.43	0.59, 3.45	0.43	1.46	0.10, 20.34	0.778
Mother's indigenous status†							
No	184/243 (75.7%)	1 (reference)					
Yes	1/1 (100%)	0.97	0.04, 24.07	0.98	0.33	0.01, 11.02	0.539
Maternal education completed							
School/ vocational	110/142 (77.5%)	1 (reference)					
Some university and above	76/103 (73.8%)	0.82	0.45, 1.48	0.51	0.74	0.36, 1.54	0.423
Mother's work status							
Full-time	79/104 (76.0%)	1 (reference)					

Variable	Infant tooth cleaning 2+ times per day (%)	Unadjusted Odds Ratio	Unadjusted 95% CI	Unadjusted P-value	Adjusted Odds Ratio	Adjusted 95% CI	Adjusted P-value
Part-time	52/69 (75.4%)	0.97	0.47, 1.96	0.93	0.88	0.41, 1.91	0.752
Self-employed	3/5 (60.0%)	0.47	0.08, 3.00	0.43	0.77	0.11, 5.26	0.785
Unemployed/ home duties/ pensioner	52/67 (77.6%)	1.09	0.53, 2.27	0.80	1.04	0.44, 2.44	0.928
<u>Source of information consulted</u>							
No information consulted							
No	141/181 (77.9%)	1 (reference)					
Yes	47/66 (71.2%)	0.70	0.37, 1.33	0.28	1.21	0.40, 3.64	0.736
Child health nurse							
No	136/179 (76.0%)	1 (reference)					
Yes	52/68 (76.5%)	1.03	0.53, 1.98	0.94	1.08	0.43, 2.73	0.870
Doctor							
No	163/218 (74.8%)	1 (reference)					
Yes	25/29 (86.2%)	2.10	0.70, 6.32	0.18	1.87	0.55, 6.30	0.313
Dietician†							
No	187/246 (76.0%)	1 (reference)					
Yes	1/1 (100%)	0.95	0.04, 23.68	0.97	0.16	0.01, 7.39	0.351
Dentist							
No	148/201 (73.6%)	1 (reference)					
Yes	40/46 (87.0%)	2.39	0.96, 5.95	0.06	3.15	1.06, 9.38	**0.039**
Mother-in-law							
No	165/219 (75.3%)	1 (reference)					
Yes	23/28 (82.1%)	1.51	0.55, 4.15	0.43	1.70	0.51, 5.67	0.387
Other female personnel (e.g. play group mums)							
No	159/208 (76.4%)	1 (reference)					
Yes	29/39 (74.4%)	0.89	0.41, 1.96	0.78	0.74	0.28, 1.92	0.530
Information pamphlets/books							
No	147/195 (75.4%)	1 (reference)					
Yes	41/52 (78.8%)	1.22	0.85, 2.55	0.60	1.84	0.66, 5.12	0.240
Pharmacist‡							
No	NA	1 (reference)					
Yes	NA	NA					
Other dental professional (e.g. dental hygienist)†							
No	187/246 (76.0%)	1 (reference)					
Yes	1/1 (100%)	0.95	0.04, 23.68	0.98	2.63	0.08, 91.13	0.594
Internet							
No	177/234 (75.6%)	1 (reference)					
Yes	11/13 (84.6%)	1.77	0.38, 8.23	0.47	2.72	0.56, 13.19	0.215
Experience from previous child							
No	183/241 (75.9%)	1 (reference)					
Yes	5/6 (83.3%)	1.58	0.18, 13.84	0.68	0.72	0.07, 7.49	0.781

Bold values represent statistical significance. CI, confidence interval.

† Firth's penalisation method applied. ‡ No data available

SUPPLEMENTARY Table S3

Results from Multivariable Logistic Regression Analysis (Unadjusted and Adjusted) on Toothpaste Usage During Deciduous Teeth Cleaning in 6-Month-Old Infants

Variable	Toothpaste used (occasionally/ regularly) when cleaning infant's teeth (%)	Unadjusted			Adjusted		
		Odds Ratio	95% CI	P-value	Odds Ratio	95% CI	P-value
All maternal characteristics							
IRSAD							
Deciles 1-2	14/66 (21.2%)	1 (reference)					
Deciles 3-5	9/73 (12.3%)	0.52	0.21, 1.30	0.16	0.26	0.08, 0.81	**0.021**
Deciles 6-8	9/58 (15.5%)	0.68	0.27, 1.72	0.42	0.69	0.24, 1.98	0.491
Deciles 9-10	9/43 (20.9%)	0.98	0.38, 2.52	0.97	1.02	0.36, 2.91	0.965
Health care card							
Yes	15/82 (18.3%)	1 (reference)					
None	20/129 (15.5%)	0.82	0.39, 1.71	0.59	1.48	0.54, 4.05	0.442
Other	2/18 (11.1%)	0.55	0.12, 2.69	0.47	1.27	0.24, 6.71	0.774
FTB Part A Eligibility							
Yes	26/145 (17.9%)	1 (reference)					
No	8/34 (23.5%)	1.41	0.57, 3.45	0.45	1.72	0.53, 5.58	0.364
Unsure	8/61 (13.1%)	0.69	0.29, 1.63	0.39	0.78	0.26, 2.35	0.659
Private insurance							
Yes	23/114 (20.2%)	1 (reference)					
No	19/124 (15.3%)	0.72	0.37, 1.39	0.33	0.59	0.25, 1.42	0.243
Mother's age at childbirth							
≤ 24 years	9/36 (25.0%)	1 (reference)					
25-34 years	28/166 (16.9%)	0.61	0.26, 1.43	0.26	0.66	0.23, 1.90	0.442
35+ years	5/39 (12.8%)	0.44	0.13, 1.47	0.18	0.47	0.11, 1.95	0.296
Mother's birth country†							
Australia, New Zealand, and UK	38/203 (18.7%)	1 (reference)					
Asia – other	2/17 (11.8%)	0.69	0.17, 2.76	0.60	2.03	0.03, 118.37	0.734
Asia – India	0/9 (0.0%)	0.23	0.01, 3.97	0.30	0.34	0.01, 60.05	0.685
Other	2/12 (16.7%)	1.02	0.25, 4.24	0.97	2.09	0.02, 194.23	0.750
Mother's primary language							
English	39/204 (19.1%)	1 (reference)					
Other	3/36 (8.3%)	0.38	0.11, 1.32	0.13	0.89	0.02, 52.17	0.955
Mother's indigenous status#							
No	42/239 (17.6%)	1 (reference)					
Yes	0/1 (0.0%)	1.55	0.06, 38.68	0.79	0.44	0.01, 18.74	0.665
Maternal education completed							
School/ vocational	33/142 (23.2%)	1 (reference)					
Some university and above	9/99 (9.1%)	0.33	0.15, 0.73	**<0.01**	0.29	0.10, 0.80	**0.017**

Variable	Toothpaste used (occasionally/ regularly) when cleaning infant's teeth (%)	Unadjusted			Adjusted		
		Odds Ratio	95% CI	P-value	Odds Ratio	95% CI	P-value
Mother's work status							
Full-time	21/103 (20.4%)	1 (reference)					
Part-time	9/66 (13.6%)	0.63	0.28, 1.46	0.28	0.32	0.11, 0.92	**0.034**
Self-employed	0/7 (0.0%)	0.25	0.01, 4.65	0.36	0.12	0.01, 2.82	0.188
Unemployed/ home duties/ pensioner	12/65 (18.5%)	0.89	0.41, 1.95	0.78	0.75	0.30, 1.92	0.553
Source of information consulted							
No information consulted							
No	31/184 (16.8%)	1 (reference)					
Yes	12/59 (20.3%)	1.26	0.59, 2.64	0.54	0.98	0.28, 3.42	0.973
Child health nurse							
No	36/172 (20.9%)	1 (reference)					
Yes	7/71 (9.9%)	0.41	0.17, 0.98	**0.05**	0.48	0.17, 1.41	0.184
Doctor							
No	37/213 (17.4%)	1 (reference)					
Yes	6/30 (20.0%)	1.18	0.45, 3.11	0.72	2.16	0.68, 6.89	0.191
Dietician†							
No	43/242 (17.8%)	1 (reference)					
Yes	0/1 (0.0%)	1.52	0.06, 31.16	0.79	3.96	0.09, 180.77	0.481
Dentist							
No	33/196 (16.8%)	1 (reference)					
Yes	10/47 (21.3%)	1.33	0.60, 2.94	0.47	1.40	0.49, 3.97	0.528
Mother-in-law							
No	38/216 (17.6%)	1 (reference)					
Yes	5/27 (18.5%)	1.06	0.37, 2.98	0.91	0.53	0.13, 2.12	0.371
Other female personnel (e.g. play group mums)							
No	34/205 (16.6%)	1 (reference)					
Yes	9/38 (23.7%)	1.56	0.67, 3.59	0.29	1.74	0.58, 5.21	0.321
Information pamphlets/books							
No	35/191 (18.3%)	1 (reference)					
Yes	8/52 (15.4%)	0.81	0.35, 1.87	0.62	0.72	0.25, 2.09	0.548
Pharmacist‡							
No	NA	1 (reference)					
Yes	NA	NA					
Other dental professional (e.g. dental hygienist)†							
No	42/242 (17.4%)	1 (reference)					
Yes	1/1 (100%)	14.15	0.56, 353.39	0.11	7.54	0.18, 321.46	0.291
Internet							
No	42/230 (18.3%)	1 (reference)					
Yes	1/13 (7.7%)	0.37	0.04, 2.94	0.35	0.24	0.03, 2.12	0.201
Experience from previous child							
No	42/237 (17.7%)	1 (reference)					

Variable	Toothpaste used (occasionally/ regularly) when cleaning infant's teeth (%)	Unadjusted			Adjusted		
		Odds Ratio	95% CI	P-value	Odds Ratio	95% CI	P-value
Yes	1/6 (16.7%)	0.92	0.10, 8.15	0.95	1.10	0.12, 10.50	0.905

Bold values represent statistical significance. CI, confidence interval.

† Firth's penalisation method applied.

‡ No data available
